# **Clinical Experience with the PillCam Patency Capsule prior to Video Capsule Endoscopy: A Real-World Experience**

**DOI:** 10.1155/2016/9657053

**Published:** 2016-01-06

**Authors:** C. Römmele, J. Brueckner, H. Messmann, S. K. Gölder

**Affiliations:** Department of Gastroenterology, Klinikum Augsburg, Stenglinstrasse 2, 86156 Augsburg, Germany

## Abstract

*Background. *In patients with known or suspected risk factors for gastrointestinal stenosis, the PillCam patency capsule (PC) is given before a video capsule endoscopy (VCE) in order to minimize the risk of capsule retention (CR). CR is considered unlikely upon excretion of the PC within 30 hours, excretion in an undamaged state after 30 hours, or radiological projection to the colon. *Methods.* We performed a retrospective analysis of 38 patients with risk factors for CR, who received a PC from 02/2013 to 04/2015 at Klinikum Augsburg. *Results.* Sixteen of our 38 patients observed a natural excretion after a mean time of 34 hours past ingestion. However, only 8 patients observed excretion within 30 hours, as recommended by the company. In 20 patients passage of the PC into the colon was shown via RFID-scan or radiological imaging (after 33 and 45 hours, resp.). Only 2 patients showed a pathologic PC result. In consequence, 32 patients received the VCE; no CR was observed. *Conclusion.* Our data indicates that a VCE could safely be performed even if the PC excretion time is longer than 30 hours and the excreted PC was not screened for damage.

## 1. Introduction

Video capsule endoscopy (VCE) is a well-established diagnostic tool in small bowel diagnostics.

The main indications are obscure gastrointestinal bleeding, suspected isolated small bowel Crohn's disease, complicated celiac disease, and surveillance in polyposis syndromes [1–4]. It is a noninvasive diagnostic tool with only rare adverse events [5]. The most important but still rare complication is capsule retention (CR). CR is defined as a remaining of the capsule in the gastrointestinal (GI) tract for longer than two weeks or the need for surgical removal due to small bowel obstruction [6]. Until now only few cases of symptomatic capsule retention have been described [7, 8]. In healthy adults the rate is almost 0%. The main risk factor for CR is known or suspected Crohn's disease with a risk of up to 13% in some studies [2, 9]. In patients with symptomatic small bowel obstruction the risk increases to over 16%. Further risk factors include NSAID enteropathy, extensive previous abdominal surgery, intestinal ischemia, volvulus, and a history of abdominal radiotherapy [6, 8, 10–12]. However, some of these may not be known before VCE.

To minimize the risk of retention in patients with risk factors for CR a PillCam patency capsule (GIVEN Imaging, Ltd., Yokneam, Israel) has been developed. The PC is a self-dissolving dummy capsule with the same size as the VCE. The capsule is made mainly of barium sulphate and lactose anhydrous. It contains a so called radiofrequency identification (RFID), which can be detected via an extracorporeal RFID scanner. 30 hours after ingestion a built-in timer opens two small holes in the capsule's surface. The digestive juice can enter the capsule and starts to dissolve the capsule, thus, preventing small bowel obstruction potentially caused by the PC. So far, only a few cases of continued retaining of the PC or temporary intestinal occlusion have been reported [13, 14].

According to the company, capsule retention of the VCE is most unlikely, if the PC has passed the intestinal tract within 30 hours or if the excreted capsule is still intact without signs of disintegration [10]. Studies showed that the sensitivity of the PC in detecting a stenosis is at least comparable to other diagnostic tools such as barium small bowel follow-through (SBFT) or CT or MRI small bowel imaging in patients with known risk factors for CR [10, 15]. A large multicenter study with 106 patients indicated that even if a stricture is radiologically diagnosed via CT or SBFT, no CR occurred during VCE when the PC was excreted undamaged after more than 30 hours.

The average total intestinal transit time of the PC in this study was 40 hours. Forty-three patients excreted the capsule within 60 hours after ingestion [12]. In this case the manufacturer demands the PC to be investigated for damage. However, the total transit time of the GI tract varies strongly depending on the general health condition, age, gender, and food consistency. Women seem to have longer transit time, whereas aging seems to accelerate the gastric and small intestinal transit [16]. The gastric passage time varies from almost instantly (fluids) to as much as 6 hours (solid and fatty foods). Passage of the small intestine usually takes about 5 hours but can take up to 7 hours in healthy adults [16]. The colon transit time again is highly variable ranging from 12 to 48 hours but can reach up to 80 hours [16].

Taking into account these highly variable physiological transit times, the cutoff of 30 hours past ingestion, as indicated by the manufacturer, can easily be exceeded in healthy subjects. It may be too short for routine clinical use.

In this retrospective study we analyzed our experiences with the APC in order to establish an algorithm suitable for a real-world scenario.

## 2. Methods

### 2.1. Study Population

All patients who had a known or suspected risk factor for CR and who received a PC from 02/2013 to 04/2015 at the Klinikum Augsburg were included in this study. Inclusion criteria were a known or suspected risk factor for a CR and the possibility of swallowing a PC. Risk factors for CR were previous abdominal operation, known or suspected Crohn's disease, long term or high dose NSAID use, suspected tumor of or in close proximity to the GI tract upon imaging, and a history of ileus or subileus.

All open abdominal surgeries except appendectomy were considered as a risk factor for CR. Laparoscopic cholecystectomy or appendectomy as well as open appendectomy was considered as a risk factor only if combined with other existing risk factors.

As for long term NSAID use, we counted the daily intake of ASS 100 for more than half a year. As for high dose NSAID we considered a daily intake of ASS 500, as well as Ibuprofen or Diclofenac for over two weeks lately.

Patients with an implantable cardioverter defibrillator (ICD) or a pacemaker were not excluded from the study although no RFID-scan could be performed.

Patients who had problems swallowing or who did not have any risk factors were not included in this study.

All patients gave written informed consent prior to the intervention. The study was conducted in accordance with the ethical principles of the Declaration of Helsinki and in compliance with good clinical practice and local regulations.

Values are expressed as mean ± standard deviation or mean (range).

### 2.2. PC Procedure

In our clinic, administration of a PC before VCE is indicated in case of any risk factors for a CR or a history of subileus/ileus. Prior to the intervention, patients were interviewed for possible swallowing problems or risk factors such as mediastinal radiotherapy or neurological disorders. Also, they were asked about a pacemaker or an ICD. All patients gave written informed consent. Before the intake of a PC, a negative scan was confirmed with the RFID scanner. Patients were allowed to eat and take their medication as usual but were asked to drink plenty. No special bowel preparations were performed. Patients who did not excrete the PC within 30 hours were scanned with the RFID scanner at 30 to 60 hours past ingestion. In case of a still positive scan, an abdominal X-ray was performed. Patients carrying a pacemaker or ICD were not scanned but X-rayed directly if the PC was not excreted within 60 hours. PCs excreted between 30 and 60 hours past ingestion were not screened for damage such as a disintegrated body. If the PC was excreted within 60 hours or radiologically projected to the colon the planned VCE was initiated.

## 3. Results

The present study is a retrospective single-center analysis at Klinikum Augsburg. We included 38 patients from 02/2013 to 04/2015, who received a PC prior to VCE because of a known or suspected risk factor for CR. The main indications for VCE were anemia/gastrointestinal bleeding (26 patients, 68%) and suspected Crohn's disease of the small intestine (10 patients, 26%). Our electronic database identified 42 patients who were scheduled for PC before VCE. Two patients had to be excluded because they could not swallow the PC. Two other patients were excluded because they intentionally aborted the diagnostic work-up and the analysis of the PC could not be completed. Thus, 38 patients met the entry criteria. The median age of the included patients was 61.5 years (range 14–88).

The patients received a PC before a VCE because of at least one risk factor for CR. Out of the 38 patients included in this study 21 patients (55%) had previous abdominal surgery, 11 patients (29%) had suspected or known Crohn's disease, 8 patients (21%) had suspected NSAID enteropathy, 4 patients (11%) had a suspected tumor in previous imaging, and 2 patients (5%) had had history of subileus/ileus.

Out of the 21 patients with previous abdominal surgery 11 patients had had multiple surgeries. Out of the 10 patients with a single abdominal surgery two had had a laparoscopic cholecystectomy, which was not considered a risk factor by itself. These patients had other risk factors as well. The remaining 8 of these patients had had additional surgeries such as small bowel resection or gastrojejunostomy.

Three of the 8 patients with suspected NSAID enteropathy had a long-time daily intake of ASS 100 mg alone. The other 5 of these patients had other accompanying risk factors for CR.

Out of the four patients with an abdominal tumor manifestation upon imaging two had a duodenal stenosis due to a malignant tumor infiltration by an unknown primary and pancreatic pseudocyst, respectively. Two other patients had a large adjacent lymphadenopathy with possible stenosis.

In conclusion, all 38 patients had documented risk factors for capsule retention. Eight patients had multiple risk factors. The patients' characteristics are summarized in Table 1.

Prior to the capsule intake, all RFID-scans were negative. Three patients were not scanned due to an implanted ICD or pacemaker.

Fourteen patients excreted the PC before the second RFID-scan at 30–60 hours past ingestion. The mean time of excretion was 33 hours (range 8–57). While 2 of the remaining patients with no excretion could not be scanned due to an implanted pacemaker or ICD, 22 patients underwent a second scan. In 6 patients the scan was negative, indicating that the PC had been excreted despite no actual observation in the stool. One patient had a positive scan but observed excretion shortly after. The remaining 17 patients underwent an abdominal X-ray at an average time of 45 hours past ingestion. In 13 out of these 17 patients the X-ray did not show the capsule or projected it to the colon, thus, indicating complete small bowel passage. One X-ray did not clearly differentiate between colon and small bowel location. This patient underwent a CT scan, which localized the capsule to the colon. Three patients' X-rays projected the PC to the small intestine. One patient observed capsule excretion shortly thereafter, at 40 hours past ingestion. The other two patients did not observe an excretion over the course of their stay. This was considered as capsule retention (CR).

One of these two patients with a pathologic PC examination was a 79-year-old woman with suspected Crohn's disease. Repeated RFID-scans and X-rays did indicate capsule retention until the 13th day after ingestion of the PC. However, the patient developed no clinical symptoms such as abdominal pain or nausea and eventual complete disintegration could be assumed. The other patient was a multimorbid 57-year-old woman who had initially been admitted to the hospital due to a urinary tract infection. She had a history of endometrial carcinoma with hysterectomy and a colorectal carcinoma with an extirpation of the rectum. A VCE was indicated due to occult gastrointestinal bleeding without sufficient explanation in upper or lower colonoscopy. A CT scan revealed a relapse of the endometrial carcinoma with pulmonic metastasis. This patient rejected any further diagnostic or therapeutic interventions and opted to be transferred to the palliative care unit.

**Figure 1:**
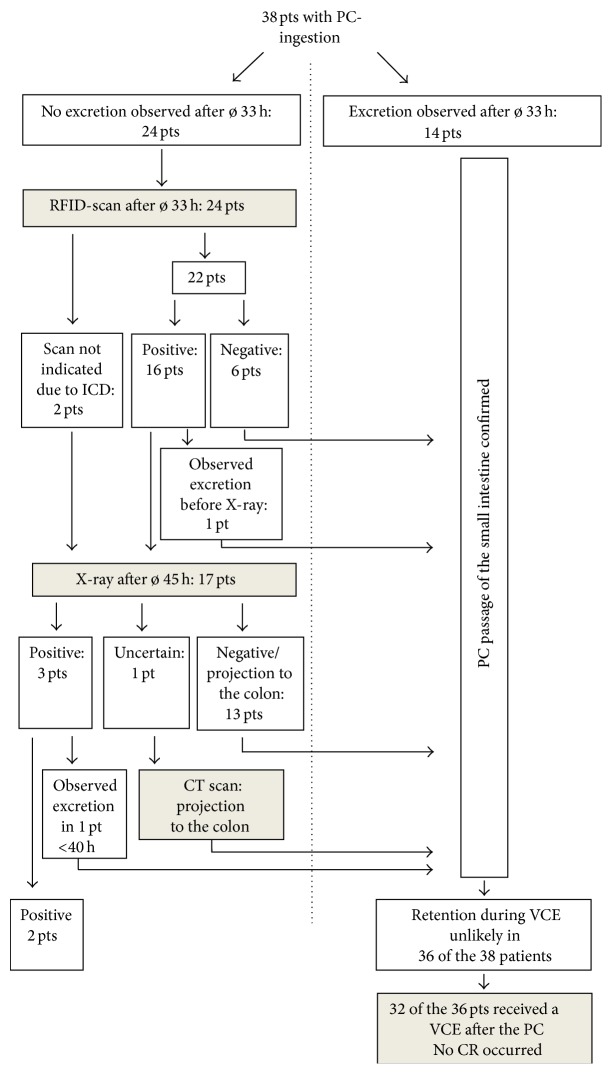
*Confirmation of patency of the small intestine by using the patency capsule (PC)*: 38 patients (pts) with an indication for VCE and risk factors for capsule retention (CR) received a PC. In 36 patients complete bowel passage could be confirmed using the PC. Sixteen patients observed natural excretion, 14 of whom before any diagnostic steps concerning possible retention. Only 2 patients showed a pathologic PC: one with suspected Crohn's disease, who gave positive RFID-scans until 13th day past ingestion, and one with a relapse of an endometrium carcinoma. PC: patency capsule, pts: patients, h: hours, VCE: video capsule endoscopy, CR: capsule retention, RFID: radiofrequency identification, and ø: mean time.

The study results are summarized in Figure 1.

In summary, 38 patients received a PC. Natural excretion was observed in 14 patients (37%) before the second RFID-scan with an average excretion time of the GI tract being 33 hours. In total 16 patients (42%) observed a natural excretion within an average passage time of 34 hours (range 8–57 hours) (Table 2). Eight patients had a total transit time of less than 30 hours. The second RFID-scan was performed after a mean time of 33 hours past ingestion. The scan was positive in 16 out of 22 patients (73%). Seventeen (45%) of the total 38 patients underwent an abdominal X-ray after a mean time of 45 hours past ingestion. This was negative in 13 patients (76%). One X-ray gave an uncertain result so a CT scan was performed. In 3 patients (11%) the X-ray projected the capsule to the small bowel, thus indicating capsule retention. One of these 3 patients observed a natural excretion after the examination.

Thirty-two (84%) of the 38 patients with PC finally underwent a VCE.

Besides the two pathologic PC examinations two other patients were afraid of capsule retention because of a longer PC passage than 30 hours. One patient developed a severe sepsis and died over the course of his stay. Another patient developed a subileus.

In total 6 patients were excluded from VCE. In the 32 VCE after PC no complication or signs for VCE retention occurred. The mean small bowel passage time of the VCE was found to be 4.4 hours. The findings of the VCE are shown in Table 2.

## 4. Discussion

The feared but rare complication of capsule endoscopy is capsule retention (CR) in the small bowel with consecutive obstruction. CR is defined as a remaining of the capsule inside the GI tract for over two weeks or the need for further steps such as surgical removal [6, 8]. Therefore the PillCam patency capsule (PC) was developed in order to test for impeded small bowel passage in patients with risk factors for CR prior to VCE. Several studies have shown that a PC can minimize the risk of CR to a high extent [8, 12, 17]. Another benefit of the PC is the lack of radiation exposure for the patient. The negative predictive value of a PC is comparable if not better compared to other diagnostic tools like small bowel follow-through (SBFT) in several retrospective studies [10, 15, 18]. Only a few cases of complications caused by the PC, such as temporary intestinal obstruction, have been reported [13, 14].

A study by Herrerias et al. in 2008 of 106 patients with known intestinal strictures could show that a VCE could be given if the PC is excreted undamaged after 30 hours [12]. Our data now show that in clinical routine only 42% of patients observe a natural excretion. Furthermore only 8 patients observed an excretion within the 30 hours past ingestion which is recommended by the company. In the other 8 patients who observed excretion after more than 30 hours, the capsule should have been examined for damage, according to the company's manual. Furthermore, 58% of patients did not detect the excreted capsule in their stool, so no inspection of the capsule would have been possible. Other studies reported a PC detection rate in the excrements of around 50% [19, 20]. In order to increase the detection rate the stool should be examined more closely; however, acceptance of such a procedure as well as the practicability in a regular clinical setting is very low.

In our study, patients were given a VCE if the PC was excreted in less than 60 hours or if the PC was radiologically projected to the colon. So 32 of the 38 patients with a PC underwent a VCE. Two patients showed a pathologic PC with no excretion within 72 hours. Two other patients rejected the VCE because their PC passage time was longer than 30 hours. One patient showed clinical signs of an ileus before administration of the VCE. Another patient died of severe sepsis probably because of a clostridium-colitis before a VCE was given. In the following 32 VCE no patient showed any signs for capsule retention. The finding that a VCE can be given, even if the PC is excreted in more than 40 hours, is consistent with findings in the literature [12, 17].

Use of bowel preparation like polyethylene glycol-based purge has been shown to improve the diagnostic gain of VCE but does not accelerate the gastric as well as small bowel capsule transit time [21, 22]. It would be interesting to know whether purging could lead to an increased capsule detection rate in the stool within the suggested 30 hours past ingestion. The official recommendation suggests a liquid diet for 20 hours and a 2-hour period of nil by mouth before and after the ingestion of the PC. Our patients were allowed to drink and eat on a regular manner. No special medication or purgative preparations were performed in accordance with other studies [20].

Limitations of this study are that it is a retrospective electronic data-based, single-center study with only 38 patients. However, a prospective randomized study would not be feasible due to ethical complications. Furthermore there is an inconsistency of time points of further diagnostic steps after the ingestion of the PC due to the clinical setting.

## 5. Conclusion

In summary, patients with known risk factors for gastrointestinal stenosis were given the PillCam patency capsule before the video capsule endoscopy in order to minimize the risk of capsule retention. Referring to the official recommendations of the company capsule retention of the video capsule endoscopy is most unlikely if the patency capsule is excreted within 30 hours, radiologically projected to the colon, or excreted in an undamaged state after 30 hours. According to our results we suggest that performing a video capsule endoscopy is safe even if the patency capsule is excreted after more than 30 hours and is not screened for damage when following our protocol. However, more data is needed.

## Figures and Tables

**Table 1:** Demographic and clinical characteristics of the study population: the main indications for the planned VCE were suspected Crohn's disease of the small intestine or gastrointestinal bleeding. All 38 included patients had at least one risk factor for a capsule retention.

Parameter	
Age [years]	61,5 [14–88]
Sex [male/female]	17/21
Indication for VCE	
Suspected CD of the small bowel	10 (26%)
Anemia/gastrointestinal bleeding	26 (68%)
Suspected tumor	2 (5%)
Documented risk factor for capsule retention	
One documented risk factor for capsule retention	8 (21%)
More than one documented risk factor for capsule retention	30 (79%)
Suspected or known Crohn's disease	11 (29%)
Previous abdominal surgery	21 (55%)
Multiple previous abdominal surgeries	11 (29%)
Previous subileus or ileus	2 (5%)
Suspected tumor in imaging	4 (11%)
High dose and/or longtime NSAID use	8 (21%)

VCE: video capsule endoscopy, CD: Crohn's disease, and NSAID: nonsteroidal anti-inflammatory drug.

**Table 2:** PC and VCE findings: transit time of the PC shown by observed natural excretion, negative RFID-scan, or projection of the PC to the colon via radiological examination. Furthermore the findings of the VCE are shown. No complications appeared during the VCE.

Totally performed PC	38
Natural excretion	
No observed excretion	22 (58%)
Observed excretion	16 (42%)
PC with transit time <30 hours	8
Mean time to observed excretion	34 hours
RFID-scan	
Total	22
Not possible (ICD, pacemaker)	2
Negative	6 (27%)
Positive	16 (73%)
Mean time performed after	33 hours
Abdominal X-ray examinations	
Total	17
Negative	13 (76%)
Uncertain, so a CT was performed afterwards	1 (6%)
Positive	3 (18%)
Mean time performed after	45 hours
Transit time of the 38 performed PCs	
PC with transit time <30 hours	8 (21%)
PC with transit time >30 hours and <72 hours	28 (74%)
PC with transit time >72 hours or retention	2 (5%)
VCE	
No VCE after PC	6 (16%)
VCE after PC	32 (84%)
VCE findings	
No pathologic finding	10 (31%)
Angiodysplasia	8 (25%)
Active bleeding	2 (6%)
Crohn's disease typical findings	7 (22%)
NSAID enteropathy typical findings	2 (6%)
Unspecific inflammation/ulcers	2 (6%)
Malignoma of the duodenum	1 (3%)
Complications	0 (0%)
Mean small bowel passage time	4.4 hours

PC: patency capsule, RFID: radiofrequency identification, VCE: video capsule endoscopy, CR: capsule retention, and NSAID: nonsteroidal anti-inflammatory drug.
